# Coronary CTA Would Facilitate Invasive Angiography in Patients With Acute Coronary Syndrome and Coronary Bypass Grafting History

**DOI:** 10.3389/fcvm.2022.751527

**Published:** 2022-03-29

**Authors:** Shaowei Ma, Ke Zhou, Yue Ma, Quanmei Ma, Yang Hou

**Affiliations:** ^1^Department of Radiology, Shengjing Hospital of China Medical University, Shenyang, China; ^2^Department of Cardiology, Shengjing Hospital of China Medical University, Shenyang, China; ^3^Department of Cardiac Surgery, Shengjing Hospital of China Medical University, Shenyang, China

**Keywords:** coronary artery disease, acute coronary syndrome, coronary bypass grafts, computed tomographic angiography, coronary angiography

## Abstract

**Background:**

The uncertainties of grafts’ ostium and patency would cause prolonged procedure/fluoroscopy time and extra contrast agent consumption of the invasive coronary angiography (ICA) in patients with coronary artery bypass grafting (CABG) history. This study was conducted to evaluate whether the identification of grafts’ ostium and patency by coronary computed tomographic angiography (CTA) could facilitate ICA procedure.

**Methods:**

Patients with acute coronary syndrome (ACS) and CABG history who underwent ICA during hospitalization were enrolled. The patients were divided into the CTA–ICA group and the direct ICA group according to whether a coronary CTA was performed before ICA. The complete direct ICA was defined by successful selective angiography of all recorded grafts. The procedure/fluoroscopy time and contrast agent consumption of ICA were compared.

**Results:**

There were 14 patients in the CTA–ICA group and 24 patients in the direct ICA group. In the direct ICA group, twelve cases were conducted complete ICA. The CTA–ICA group had reduced procedure time (17.8 ± 7.1 vs. 25.9 ± 15.4 min, *p* = 0.03) and fluoroscopy time (fluor-time; 4.6 ± 2.3 vs. 9.8 ± 5.3 min, *p* < 0.01), and less contrast agent consumption (30.4 ± 5.6 vs. 49.8 ± 20.9 ml, *p* < 0.01) than the direct ICA group. In a subgroup analysis, the incomplete direct ICA had the longest procedure time (32.8 ± 16.5 min) or fluor-time (12.0 ± 5.5 min) and the most contrast agent consumption (58.3 ± 25.8 ml), whereas the difference between CTA–ICA and complete direct ICA groups was non-significant.

**Conclusion:**

The CTA would facilitate invasive angiography in patients with CABG by reducing procedure/fluoroscopy time and contrast agent consumption.

## Introduction

Coronary revascularization could effectively recover the myocardial blood supply and improve the prognosis of patients with severe coronary artery disease (CAD) (1). Coronary artery bypass grafting (CABG) is preferred for patients with diabetes mellitus (DM) or/and high anatomical complexity (2–6). Although the patency rates of left internal mammary artery (LIMA) grafts maintain 90% in over 10-year follow-up (7, 8), saphenous vein grafts (SVGs) failed in 40-50% of treated patients (9). Acute coronary syndrome (ACS) would occur if the grafts failed or/and native coronary atherosclerosis progressed.

Coronary anatomy assessing is an indispensable step for the management of ACS. As a non-invasive method, coronary computed tomographic angiography (CTA) has a high negative predictive value (NPV) to exclude ACS by excluding CAD (10). However, the correlation between CTA and invasive coronary angiography (ICA) anatomical assessment was moderate for multivessel evaluation (11), and it was not clearly recommended for patients with ACS and CABG history (10). The invasive angiography combined with intracoronary imaging or/and functional assessing is highly recommended in the management of patients with ACS and multivessel/left main lesions (1, 10). Thus, although CTA could be helpful for long-term risk stratification of patients with CABG (12), the ICA might be the first choice for anatomic assessment in some patients with ACS and CABG history. Unfortunately, the uncertainties of the patency and ostium of the bypassing grafts (especially SVG) might perplex the ICA procedure even for experienced operators (such as prolonged procedure/fluoroscopy time, increased contrast agent consumption, and a higher incomplete ICA rate). Previous study indicated that the CTA–ICA pattern could facilitate the ICA procedure for the overall patients with CABG (13). However, the evidence for the improvement of ICA procedure by the CTA–ICA pattern in patients with ACS who might had a higher tendency to direct ICA was lacking.

This study was conducted to evaluate whether the coronary CTA performed before ICA (the CTA–ICA pattern), in comparison with direct ICA, could facilitate the invasive procedure by reducing procedure/fluoroscopy time and contrast agent consumption in patients with ACS and prior CABG history.

## Materials and Methods

### Study Design

This study included data of patients scanned between January 2017 and December 2020 in Shengjing Hospital of China Medical University, Shenyang, China. The inclusion criteria were: (i) patient with a previous CABG history and admitted for ACS included unstable angina (UA), non-ST-segment elevation myocardial infarction (NSTEMI), and ST-segment elevation myocardial infarction (STEMI) (10, 14); and (ii) the ICA was performed during hospitalization. All enrolled patients were divided into the CTA–ICA group or direct ICA group according to whether a coronary CTA was performed before ICA or not.

This study was approved by the institutional review board of Shengjing Hospital of China Medical University. Because it was an observational study and the examination was necessary for clinical diagnosis of the subjects, written informed consent was waived.

### Invasive Coronary Angiography

Selectively ICA was performed according to the standard protocol. All the operators had an over 3-year experience for ICA (at least 300 cases per year as a solo operator). The right radial artery was the first choice of arterial approach if there was no need for LIMA angiography. The left radial artery or the right femoral artery was the standard approach if LIMA angiography was necessary. The catheters used in ICA were radial TIG (Terumo Corporation, Tokyo, Japan) and Judkins left/right or Amplatz (Cordis Corporation, Miami Lakes, United States). For patients in the direct ICA group, the ICA procedures were divided into complete or incomplete angiography. Complete direct ICA was defined by successful selective angiography of all native coronary arteries and bypass grafts (based on surgical recordings). If there was any unsuccessful selective angiography of native coronary arteries or bypass graft (including occlusive grafts), the procedure was recorded as incomplete direct ICA. An ascending aortography (contrast agent volume: 30–40 ml, injection velocity: 15 ml/s, and pressure: 800 pounds per square inch) was performed with a 6-Fr pigtail catheter (Cordis Corporation, Miami Lakes, United States) when necessary.

**FIGURE 1:**
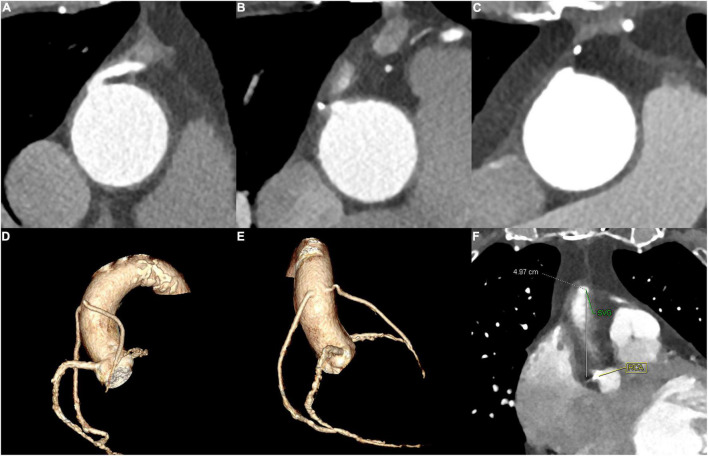
The representative cases for SVG identified by CTA. **(A)** and **(B)** A 65–70-year-old patient of the CTA–ICA group who had two SVG. **(A)** The patency of one SVG and **(B)** the occlusion of the other SVG. **(C)** A 60–65-year-old patient who had an incomplete direct ICA and ascending aortography, a subsequent CTA successfully recognized the graft’s occlusion. **(D–F)** A 70–75-year-old patient of the CTA–ICA group: the LAO and RAO of the three-dimensional reconstruction are shown in **(D)** and **(E)**, and the distance between SVG and RCA ostium was measured **(F)**; therefore, the operator could locate the grafts’ ostium with the RCA as a reference. SVG, saphenous vein grafts; CTA, computed tomographic angiography; ICA, invasive coronary angiography; RCA, right coronary artery; LAO, left anterior oblique; RAO, right anterior oblique.

If coronary CTA images were available before ICA procedure, the operators would carefully read the CTA images to identify the patency and locate the ostium of the grafts. The representative cases are shown in Figure 1.

### Recordings of Invasive Coronary Angiography Characteristics

The ICA procedure time, fluoroscopy time (fluor-time), and contrast agent consumption were recorded, and the number of catheters used during the procedures. The procedure time was defined from the successful establishing of arterial approach to the end of the angiography, the fluor-time was automatically calculated by the software of digital subtraction angiography (DSA) device (Bransist Safire, Shimadzu Corporation, Japan). The contrast agent consumption was estimated at the end of angiography, whereas the contrast agent used for intervention therapy was not included.

### CT Acquisition and Reconstruction

A coronary CTA was performed at least 24 h before ICA according to the clinicians’ decision (mainly based on their personal experience for ICA and the availability of the surgical records for CABG). All CTA scanning was carried out in accordance with the guidelines recommended by the Society of Cardiovascular Computed Tomography (15). The scans were performed on 256-slice multidetector CT (Brilliance iCT, Philips Healthcare, Cleveland, OH, United States) with ECG gating. The main scanning parameters were as follows: tube potential = 120 kVp; effective tube current–time product = 105 mAs; slice thickness = 0.9 mm; and increment = 0.45 mm. The imaging trigger was centered around a physiological cardiac phase of ventricular diastasis corresponding to 75% of the R–R interval. Contrast media Visipaque (Iodixanol 270; GE Healthcare, Ireland) was injected with a flow rate of 4.5 ml/s (< 80 kg body weight) or 5 ml/s (≥ 80 kg body weight) followed by a 20-ml saline flush. The total amount of contrast media was patient weight × 0.7 ml/kg. The oral β-receptor blockers were administered before scanning when necessary.

### Statistical Analysis

Data were analyzed using commercially available software (SPSS version 20.0, United States). The continuous variables were described by mean ± SD. Categorical variable was expressed as percentage.

Independent-sample *t*-test or chi-square test was applied to compare the difference of patients’ characteristics, and the difference of ICA procedure time, fluor-time, and contrast agent consumption between CTA–ICA and direct ICA groups. One-way ANOVA was used to evaluate the difference between CTA–ICA and complete and incomplete direct ICA groups. The *p*-value < 0.05 was considered statistically significant.

## Results

### Patients’ Characteristics

**FIGURE 2:**
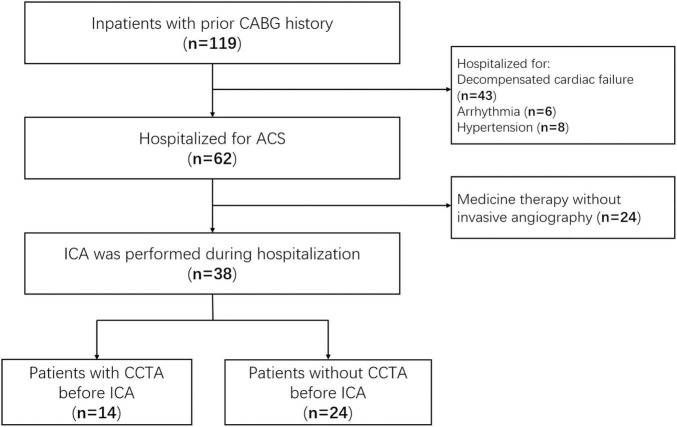
Study flow diagram. CABG, coronary artery bypass grafting; ACS, acute coronary syndrome; ICA, invasive coronary angiography; CCTA, coronary computed tomographic angiography.

A total of 62 patients with CABG were hospitalized for ACS during the screen period, 38 of them underwent ICA and enrolled in this study as shown in Figure 2. Fourteen patients were performed coronary CTA before ICA. There was no significant difference in the Global Registry of Acute Coronary Events (GRACE) score between the two groups (119.2 ± 23.8 vs. 112.7 ± 17.2, *p* = 0.36). There were 31 coronary bypassing grafts in the CTA–ICA group, and the occlusion was identified by CTA in 4 of them. All the rest 27 non-occlusive grafts had a successful selective angiography during ICA procedure. Twelve cases were defined as incomplete ICA in the direct ICA group. Six of these patients with incomplete ICA underwent ascending aortography, and four of them were performed coronary CTA after the ICA (three patients underwent a second invasive procedure for interventional therapy after coronary CTA). Moreover, one patient underwent an unnecessary LIMA angiography in the direct ICA group (the LIMA was not used for coronary bypassing). The details of patients’ characteristics are shown in Table 1.

**TABLE 1:** Patients characteristics in the CTA–ICA and direct ICA groups.

	CTA–ICA (*N* = 14)	Direct ICA (*N* = 24)	*p-*value
Age (year)	66.2	66.3	0.93
Gender (male,%)	9 (64.3%)	18 (75.0%)	0.74
BMI	27.0 ± 2.7	24.9 ± 2.0	0.02
Diagnosis: UA	7 (50%)	15 (62.5%)	0.49
NSTEMI	7 (50%)	8 (33.3%)	
STEMI	0	1 (4%)	
Killip class 1	10	20	0.37
Killip class 2	4	3	
Killip class 3	0	1	
Killip class 4	0	0	
Duration of CABG to ICA (Year)	6.3	6.8	0.73
Hypertension (%)	11 (78.6%)	18 (75.0%)	0.88
DM (%)	6 (42.9%)	13 (54.2%)	0.74
Current Smoker (%)	4 (28.6%)	8 (33.3%)	0.95
LDL-C (mmol/L)	2.5 ± 0.7	2.7 ± 1.5	0.71
HbA1c	6.9 ± 1.4	6.8 ± 1.2	0.92
hs-cTn > 5-fold UL	5 (35.7%)	7 (29.2%)	0.68
Crea Pre-ICA (μmol/L)	77.1 ± 28.8	78.3 ± 22.9	0.89
Crea Post-ICA (μmol/L)	80.9 ± 24.2	78.2 ± 22.7	0.75
CrCl (ml/min)	93.6 ± 35.9	80.8 ± 17.0	0.18
LVEF (%)	60.1 ± 3.6	56.4 ± 8.8	0.08
GRACE Score	119.2 ± 23.8	112.7 ± 17.2	0.36
Oral medication			
Aspirin + Clopidogrel	11 (78.6%)	15 (62.5%)	0.31
Aspirin + Ticagrelor	3 (21,4%)	9 (37.5%)	0.31
Statins	14 (100%)	24 (100%)	-
β receptor blockers	12 (85.7%)	19 (79.2%)	0.69
ACEI/ARB	9 (64.3%)	17 (70.8%)	0.68
Access (N)			
Right radial artery	6	8	0.37
Left radial artery	8	13	
Right femoral artery	0	3	
Number of grafts (N)	2.1 ± 0.7	2.4 ± 0.6	0.3
Grafts identified by ICA (N)	1.9 ± 0.7	1.8 ± 0.6	0.68
Catheters (N)	1.8 ± 0.8	2.2 ± 0.9	0.17
TIG	12	12	0.06
Judkins left	5	13	0.45
Judkins right	7	17	0.15
Amplatz	1	5	0.51
Pigtail	0	6	0.11
Ascending aortography	0	6	0.11

*CTA, computed tomographic angiography; ICA, invasive coronary angiography; BMI, body mass index; UA, unstable angina; NSTEMI, non-ST-segment elevation myocardial infarction; STEMI, ST-segment elevation myocardial infarction; DM, diabetes mellitus; LDL-C, low-density lipoprotein cholesterol; HbA1c, glycosylated hemoglobin; hs-cTn, high-sensitivity cardiac troponin; UL, upper reference limit; Crea, serum creatinine; CrCl, estimated creatinine clearance rate, CrCl = [(140–age) × weight]/(72 × Cr)] for men, for women the result was multiplied by 0.85; LVEF, left ventricular ejection fraction; ACEI, angiotensin converting enzyme inhibitor; ARB, angiotensin receptor blockers; TIG, radial multifunction catheter; fluor-time, fluoroscopy time.*

### The Comparison Between CTA–ICA and Direct ICA Groups

**FIGURE 3:**
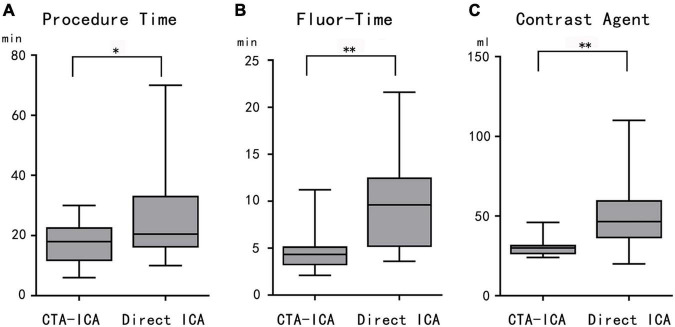
The comparison between CTA–ICA and direct ICA groups. The procedure time **(A)**, fluor-time **(B)**, and contrast agent consumption **(C)** of the CTA–ICA group are significantly decreased comparing with the direct ICA group. CTA, computed tomographic angiography; ICA, invasive coronary angiography; fluor-time, fluoroscopy time; *p < 0.05, **p < 0.01.

All patients in the CTA–ICA group had radial artery access (right: 6, left: 8), whereas the accesses of the direct ICA group were 21 cases with radial artery (right: 8, left: 13) and 3 cases with the right femoral artery. In comparison with the direct ICA group, the CTA–ICA group had much shorter ICA procedure time (17.8 ± 7.1 vs. 25.9 ± 15.4 min, difference: − 8.1 min, 95% confidence interval [CI]: − 15.6 to − 0.7 min, *p* = 0.03, Figure 3A), fluor-time (4.6 ± 2.3 vs. 9.8 ± 5.3 min, difference: − 5.2 min, 95% CI: − 7.8 to − 2.7 min, *p* < 0.01, Figure 3B) and reduced contrast agent consumption during ICA (30.4 ± 5.6 vs. 49.8 ± 20.9 ml, difference: − 19.3 ml, 95% CI: − 28.6 to − 10.1 ml, *p* < 0.01, Figure 3C).

### The Comparison Among CTA-ICA, Complete and Incomplete Direct ICA Groups

**FIGURE 4:**
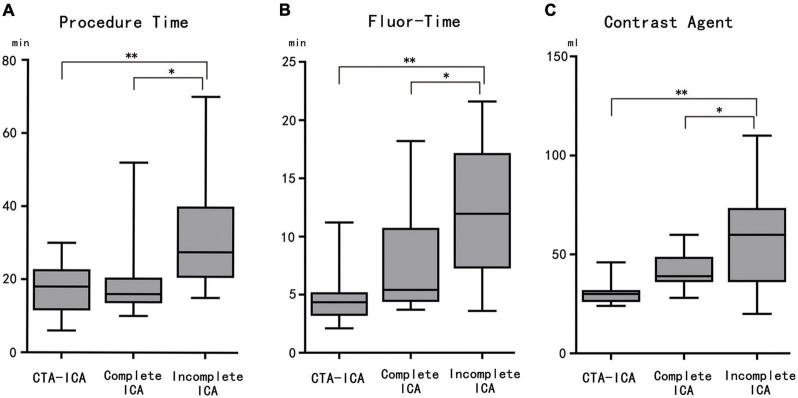
The comparison among CTA–ICA and complete and incomplete direct ICA groups. The procedure time **(A)**, fluor-time **(B)**, and contrast agent consumption **(C)** of the incomplete direct ICA group are significantly increased comparing with the other two groups. CTA, computed tomographic angiography; ICA, invasive coronary angiography; fluor-time, fluoroscopy time; *p < 0.05, **p < 0.01.

To investigate whether the completeness would affect the direct ICA procedure, a subgroup analysis was performed (Table 2). Results showed that in comparison with the CTA–ICA group, the procedure/fluoroscopy time and contrast agent consumption were non-significantly increased in the complete direct ICA group (procedure time: 17.8 ± 7.1 vs. 19.1 ± 11.1 min, *p* = 0.95; fluor-time: 4.6 ± 2.3 vs. 7.6 ± 4.4 min, *p* = 0.17; contrast agent: 30.4 ± 5.6 vs. 41.2 ± 9.3 min, *p* = 0.21). However, significant increase of procedure/fluoroscopy time and contrast agent consumption was observed in the incomplete direct ICA group than the CTA–ICA group (procedure time: 17.8 ± 7.1 vs. 32.8 ± 16.5 min, *p* < 0.01; fluor-time: 4.6 ± 2.3 vs. 12.0 ± 5.5 min, *p* < 0.01; contrast agent: 30.4 ± 5.6 vs. 58.3 ± 25.8 min, *p* < 0.01), Figure 4.

**TABLE 2:** The comparison among CTA-ICA, complete and incomplete direct ICA groups.

	CTA–ICA (group a, *N* = 14)	Complete direct ICA (group b, *N* = 12)	Incomplete direct ICA (group c, *N* = 12)
Crea Pre-ICA (mmol/L)	77.1 ± 28.8	73.5 ± 13.4	83.6 ± 30.2
Crea Post-ICA (mmol/L)	80.6 ± 24.2	70.9 ± 13.4	87.1 ± 29.0
Number of grafts (N)	2.1 ± 0.7	2.1 ± 0.7	2.6 ± 0.5
Grafts identified by ICA (N)	1.9 ± 0.7	2.1 ± 0.7_c_*_	1.6 ± 0.5
Number of catheters	1.8 ± 0.8	1.8 ± 0.6	2.6 ± 1.1

CTA, computed tomographic angiography; ICA, invasive coronary angiography; Crea, serum creatinine; fluor-time, fluoroscopy time; *p < 0.05.

## Discussion

The present study showed that coronary CTA could provide sufficient information of the coronary bypassing grafts (particularly the patency/occlusion). Furthermore, the CTA–ICA pattern significantly shortened the procedure/fluoroscopy time, and decreased the contrast agent injection of the invasive angiography. Thereby, our results supported the complementary function between coronary CTA and ICA in complex coronary anatomic assessment.

A recent published study indicated that the CTA–ICA pattern could significantly shorten procedure/fluoroscopy time and decrease the contrast agent consumption (13). However, they did not further analyze the reason for these improvements. Accordingly, a subgroup analysis was conducted in the present study. The results indicated that the incomplete direct ICA group had the longest procedure/fluoroscopy time and the largest contrast agent consumption, whereas the differences between CTA–ICA and complete direct ICA groups were non-significant. Therefore, we assumed that the benefits of coronary CTA for graft angiography were mainly resulted from the recognition of graft occlusion before the invasive procedure. As the benefit of locating grafts’ ostium, it might be varied among operators with different CTA image reading and catheter operating skills. This might be the reason for the non-significant improvement of the CTA–ICA group in comparison with the complete direct ICA group. The 3-dimensional image fusion technique would be helpful for grafts locating during ICA (16). However, as this image fusion method requires additional equipment and software, its utilization might be limited.

With the improvements of CTA scanning and image reconstruction techniques, the coronary CTA could provide detailed evaluation coronary bypassing grafts (17–19). And the experts of Society of Cardiovascular Computed Tomography (SCCT) suggested that it was appropriate to perform CTA for patients with prior CABG, particularly when graft patency is the primary objective (20). However, the correlation between CTA and ICA anatomical assessment was moderate for multivessel evaluation (11). Moreover, the inaccurate visualization of distal anastomoses and clip artifacts might affect the diagnostic accuracy of CTA (21). Study had indicated that patients with prior CABG who received percutaneous coronary intervention (PCI) had better in-hospital clinical outcomes compared with those who received medical management alone (22). Thus, the ICA still has an edge for patients with prior CABG.

Unfortunately, patients with prior CABG had a lower rate for coronary angiography and PCI (22). Meanwhile, previous study had indicated that patients with coronary bypass grafts had a higher incomplete ICA rate than patients without CABG history, and a subsequent coronary CTA was in demand to confirm the graft’s occlusion (23). Regrettably, the treatment decision-making might be delayed, and an additional invasive procedure was needed in the ICA–CTA–PCI pattern. The proportion of incomplete ICA was up to 50% in the direct ICA group of present study. In this study, a broad definition of incomplete ICA was applied, the incomplete ICA was defined by unsuccessful selective angiography of any recorded bypass grafts even when the graft was occlusive. It was reported that SVG might fail in 40%-50% of overall patients with CABG (9), and Shavadia et al. indicated that there were 27.0% of arterial and 34.5% of vein graft failure in symptomatic patients (64.4% with ACS) with prior CABG history (24). Thus, it might be reasonable that we had a relatively high rate of incomplete direct ICA in patients with ACS.

Jones et al. applied the similar definition for incomplete ICA and reported a rate of 25.6% incomplete angiography in overall patients with CABG (13). However, as the patients included in this study were all admitted for ACS, the incomplete ICA rate was even higher. As indicated by present study, the incomplete direct ICA could lead to significant longer procedure time. It had been demonstrated that procedural time was a powerful predictor of ICA complications in patients with prior CABG (25). Although radiopaque bypass graft marker and sternal wire code could be helpful for grafts locating during ICA (26, 27), they might not help to reduce the procedure time and contrast media consumption of incomplete ICA by identifying graft occlusion. The ascending aortography could be used for non-selective CABG angiography in the incomplete ICA cases, but it would increase the cardiac afterload and contrast agent consumption. Furthermore, the increasing contrast media volume of PCI had been identified as an independent factor for contrast-induced nephropathy (CIN) (28). Thus, the patients with ACS who have a high possibility of graft failure might acquire more benefit from the CTA–ICA pattern.

According to the present results, a coronary CTA performed before ICA could provide intuitionistic and detailed anatomic information of the coronary bypassing grafts (patency and ostium location). Therefore, the following invasive procedure would be safer and more effective with this confirmed information. With the assistance of CTA, the ICA procedure time (the mean time for the CTA–ICA group was only 69% of the direct ICA group) and fluor-time (the mean time for the CTA–ICA group was less than half of the direct ICA group) could be significant shortened, which would not only improve patients’ comfort but also reduce the radiation exposure and complications of ICA (the patient’s total radiation exposure was not increased by a previous CTA scanning (13)). Furthermore, the additional invasive procedure for interventional therapy might be avoided by changing the ICA–CTA–PCI pattern with CTA–ICA/PCI. However, whether the CTA–ICA pattern should be applied for urgent situations in patients with high GRACE scores or STEMI was a dilemma. Although the CTA–ICA pattern would lead to the delay of ICA initiation, the direct ICA might result in inappropriate revascularization caused by incomplete graft angiography (29). Further investigation was in demanded for this complex issue.

As a preliminary study, the limitations were as follows: first, this was a single-center study with a small sample size, the difference caused by different operators could not be avoided; Second, radiation doses of ICA procedure were not available due to DSA equipment limitations; thus, the patients’ total radiation exposure could not be analyzed; third, patients included in this study were mainly with low-intermediate risk and their cardiac troponin were not intensively monitored, thus the effect of myocardial enzymes on the CTA–ICA pattern selection and whether the CTA–ICA pattern should be used in patients with high GRACE scores were in demand for further investigation. Finally, the information of CABG conducted in other medical centers was briefly recorded without the original surgical record document, the selection bias might exist in the CTA–ICA/direct ICA pattern selection.

## Conclusion

The CTA–ICA pattern for patients with ACS and CABG history could significantly reduce the operating/fluoroscopy time and contrast agent consumption during the ICA procedure. Therefore, the CTA–ICA pattern might help to remarkably improve the efficiency and safety of invasive procedure in such patients.

## Data Availability Statement

The original contributions presented in the study are included in the article/supplementary material, further inquiries can be directed to the corresponding author/s.

## Ethics Statement

The studies involving human participants were reviewed and approved by the Institutional Review Board of Shengjing Hospital of China Medical University. Written informed consent for participation was not required for this study in accordance with the national legislation and the institutional requirements.

## Author Contributions

SM and YH contributed to conception and design of the study. SM, KZ, YM, and QM conducted the data collection and the statistical analysis. SM wrote the first draft of the manuscript. All authors contributed to manuscript revision, read, and approved the submitted version.

## Conflict of Interest

The authors declare that the research was conducted in the absence of any commercial or financial relationships that could be construed as a potential conflict of interest.

## Publisher’s Note

All claims expressed in this article are solely those of the authors and do not necessarily represent those of their affiliated organizations, or those of the publisher, the editors and the reviewers. Any product that may be evaluated in this article, or claim that may be made by its manufacturer, is not guaranteed or endorsed by the publisher.
